# High infliximab trough concentrations are associated with sustained histologic remission in inflammatory bowel disease: a prospective cohort study

**DOI:** 10.1186/s12876-021-01650-7

**Published:** 2021-02-18

**Authors:** Aze Wilson, Bethany Choi, Michael Sey, Terry Ponich, Melanie Beaton, Richard B. Kim

**Affiliations:** 1 Divisions of Clinical Pharmacology, Department of Medicine, Western University, 339 A Wilson 339 Windermere Road A10-221a, London, ON N6A 5A5 Canada; 2 Divisions of Gastroenterology, Department of Medicine, Western University, 339 Windermere Rd, London, ON N6A 5A5 Canada; 3 Department of Physiology and Pharmacology, Western University, Medical Sciences Building, Rm 216, London, ON N6A 5C1 Canada

**Keywords:** Inflammatory bowel disease, Histologic remission, Inflixmab trough concentrations

## Abstract

**Background:**

The threshold concentration of infliximab during maintenance therapy has not been well-defined in relation to histologic remission. The aim of the study is to dentify the maintenance-phase infliximab concentration associated with histologic remission in inflammatory bowel disease patients (IBD).

**Methods:**

A prospective cohort study was carried out in 104 IBD patients seen at a tertiary care centre in London, Canada. Infliximab trough concentrations were collected during the maintenance phase of treatment and compared between participants with and without evidence of histologic remission. Participants were additionally evaluated for sustained histologic remission, and relapse to active disease.

**Results:**

Participants in histologic remission attained higher mean concentrations of infliximab during the maintenance phase (10.34 ± 0.69 μg/ml) compared to those with persistent disease activity (6.23 ± 0.67 μg/ml, p-value < 0.0001). Additionally, during the maintenance phase, sustained histologic remission was also associated with a higher mean concentration of infliximab (10.81 ± 5.46 μg/ml) compared to those who relapsed to active disease (5.68 ± 3.70, p < 0.001). Overall, participants with a mean infliximab trough concentration greater than 8ug/ml were more likely to have histologic remission (area under the receiver operating characteristic curve, AUROC = 0.72, 95%CI = 0.65–0.84, p < 0.0001) and sustained histologic remission (AUC = 0.77, 95%CI = 0.63–0.91, p = 0.002).

**Conclusion:**

Maintenance-phase infliximab trough concentrations greater than 8 μg/ml, which is higher than the currently recommended target concentration, are highly associated with histologic remission and sustained histologic remission.

## Background

Infliximab therapy has been a mainstay of inflammatory bowel disease (IBD) management even as the landscape of therapeutic monoclonal antibodies continues to evolve. Infliximab remains a first line treatment for moderate-to-severe Crohn's disease (CD) and ulcerative colitis (UC) as recommended by international IBD guidelines and is the sole biologic therapy available as rescue therapy for the hospitalized IBD patient [1–5].

Infliximab is a chimeric IgG1 immunoglobulin directed against the pro-inflammatory cytokine, tumour necrosis factor-α (TNF). The efficacy of infliximab in CD and UC has been demonstrated in landmark trials: ACCENT and ACT respectively [6, 7]. High infliximab trough concentrations over time appear to be an important determinant of infliximab efficacy [8–10]. Infliximab efficacy is defined by its ability to induce and maintain disease remission. The majority of clinical trials evaluating infliximab defined efficacy as clinical remission, which is a lack of symptoms based on validated clinical scoring indices. Clinical symptoms, however, do not always correlate with intestinal disease activity [11, 12]. Targeting symptomatic endpoints has not been predictive of hard outcomes such as need-for-surgical intervention [13–15]. As a result, there has been an increasing shift towards using improvement in endoscopic appearance, also known as “mucosal healing”, as a more stringent outcome for drug effectiveness [16, 17]. Interestingly, ongoing histologic disease activity, defined as persistent architectural distortions and/or increased inflammatory infiltrates in the lamina propria or epithelium, exists in a subset of individuals with endoscopically quiescent disease [18–20]. This has lead many clinicians and regulatory authorities to speculate whether or not treating to mucosal healing should equate to both endoscopic *and* histologic remission [16, 21, 22]. This is supported by the fact that histologic remission is associated with a reduced risk of relapse, surgery, hospitalization and colon cancer in UC[23]. Data are less available for CD; however, limited studies suggest that histologic remission is associated with reduced risk of relapse and increased rates of corticosteroid-free remission[24]. Several studies, including post-hoc analyses of several key clinical trials, have confirmed that infliximab is associated with the induction of histologic remission in both CD and UC [6, 25, 26].

Several observational studies have investigated the threshold infliximab concentrations associated with increasingly refined definitions of remission. The majority of the published data pertain to infliximab concentrations *and* clinical remission and indicate that maintenance-phase infliximab trough concentrations greater than 3.1ug/ml are associated with clinical remission [10]. Consequently, American IBD guidelines recommend that clinicians target infliximab concentrations greater than 5ug/ml[27] Much less is known in terms of infliximab concentration targets for achieving endoscopic remission or histologic remission; however, according to the studies that do exist, higher infliximab concentrations are needed with increasingly stringent therapeutic targets[9, 28, 29] Thus, given the small number of studies evaluating histologic remission and infliximab trough concentrations, we aimed to evaluate the association between histologic remission and mean infliximab trough concentrations during the maintenance phase of therapy in an IBD population.

## Methods

### Participants

A prospective cohort study was carried out at two tertiary-care hospitals, both affiliated with Western University (London, Canada). Recruitment took place between November 1, 2015 and December 1, 2018. Eligible participants were adults with a histopathologic diagnosis of either UC or CD treated with infliximab within the maintenance phase of treatment, defined as a minimum exposure of 14 weeks. In addition to infliximab, the use of a combined immunomodulator, glucocorticoid or 5-aminosalicylate was permitted and at the discretion of the treating physician. Infliximab dose adjustments including the use of high-dose infliximab (defined as infliximab dose > 5 mg/kg or a dosing interval less than 8 weeks) were allowed. Included participants also had to have record of two colonoscopies completed during the maintenance phase of infliximab treatment. Participants were excluded if they were in the induction phase of infliximab treatment, if they did not have two endoscopic assessments, if there was no available histopathology for assessment of disease activity or if there were less than three infliximab concentrations available. The study protocol was approved by the Western University Health Sciences Research Ethics Board. All study subjects provided written informed consent. All procedures performed were in accordance with the ethical standards of the Western University Health Sciences Research Ethics Board and with the 1964 Helsinki declaration and its later amendments or comparable ethical standards.

### Data collection

Participants were assessed at consecutive infliximab infusions up to a maximum of 7 infusions. Patients who discontinued infliximab prior to the 7th infusion were administratively censored. Data collected on all participants included age, sex, weight, IBD diagnosis, disease duration and location, smoking history, IBD medication exposures and responses, hospitalizations, history of surgical resection, and symptoms based on the clinical scoring index, HBI and partial Mayo score at the time of infusion [30, 31]. Blood samples were drawn at each infusion for infliximab trough concentration determination. Colonoscopy and histopathology reports were reviewed prior to study enrollment (baseline colonoscopy). Participant follow-up was continued up to one year following the period of blood collection (up to 7 infusions), with patient charts reviewed monthly for the confirmation of completion of a second colonoscopy (follow-up colonoscopy) with available histopathology.

Participants with UC were required to have biopsies obtained from at least the rectum, sigmoid, descending and ascending colon. Participants with CD were required to have biopsies obtained from all segments where disease had been previously described (ileum, ileo-colonic, or colonic) with individuals with colonic disease having biopsies taken from multiple colonic sites.

### Quantification of infliximab trough concentrations and antibodies to infliximab (ATI)

Blood samples were drawn immediately prior to consecutive infliximab infusions for all participants. Plasma was immediately separated from whole blood and stored at -80^◦^C until further use. Infliximab trough concentrations were measured using an enzyme-linked immunosorbent assay (ELISA) by a colorimetric detection method (Abcam, Cambridge UK) as detailed in the manufacturer's protocol. All samples were assessed in duplicate and the lower limit of detection was 0.1 μg/ml. For samples falling below the lower limit of detection, a follow-up assessment for detection of ATI was performed using an anti-infliximab ELISA (Abcam, Cambridge UK). Samples were assessed in duplicate. The lower limit of detection for ATI by this method was 62 ng/ml.

### Study outcomes

The objective of this study was to test the hypothesis that maintenance-phase infliximab concentrations higher than what is recommended in clinical guidelines are associated with histologic remission in IBD [27]. The mean infliximab concentration with standard deviation was calculated for each participant using all drug concentrations recorded during the study period. The primary endpoint was histologic remission, defined as an absence of active chronic inflammation and/or deemed "normal" by an expert GI pathologist. Secondary outcomes included sustained histologic remission (histologic remission documented at both the baseline and follow-up colonoscopies), histologic disease relapse (defined as the presence of chronic, active inflammation on the follow-up colonoscopy), new histologic remission (defined as the presence of chronic, active inflammation at the baseline colonoscopy and histologic remission on the follow-up colonoscopy) and failure to recapture response (secondary non-responders with histologic disease activity at both colonoscopies). Other endpoints included determination of an infliximab concentration threshold associated with histologic remission and sustained histologic remission. Intra-patient variation (IPV) (calculated by dividing the standard deviation by the mean infliximab trough concentration) in infliximab trough concentrations based on histologic remission was also assessed.

### Statistical analyses

Statistical analyses were performed using Graphpad Prism version 5 and SPSS version 17.1 statistical softwares. Descriptive statistics were used to summarize data obtained for the cohort including frequency distributions for categorical variables and medians with interquartile ranges (IQR) or ranges for continuous variables.

The Shapiro–Wilk test was used to test the normality of distribution for continuous outcomes. Based on this, a Mann–Whitney U test was selected to compare the mean infliximab trough concentrations between participants based on histologic remission at the time of follow-up colonoscopy. A Kruskal–Wallis test using Dunn’s multiple comparison test was used to compare the IPV and mean infliximab trough concentrations between relapsers versus those with sustained remission versus those achieving a new remission. A p-value < 0.05 was considered significant.

Receiver-operating characteristic (ROC) analysis was used to identify the threshold mean infliximab concentration associated with histologic remission and sustained histologic remission. The best threshold was identified by the Youden index [32] Area under the ROC curve, in addition to the sensitivity and specificity, for the optimal thresholds are reported.

Additionally, the effect of the mean infliximab trough concentration thresholds identified on ROC analysis for histologic remission and sustained histologic remission were assessed using a multivariable logistic regression. Additional covariates included age, sex, weight, IBD diagnosis, disease duration, presence of anti-infliximab antibodies, co-immunosuppression with one of azathioprine or methotrexate, mean infliximab dose and interval. A p-value < 0.05 was considered significant.

Lastly, to test the hypothesis that there is a threshold infliximab concentration beyond which there is no further gain in histologic remission, an incremental gain analysis was conducted [33]

## Results

**Fig. 1:**
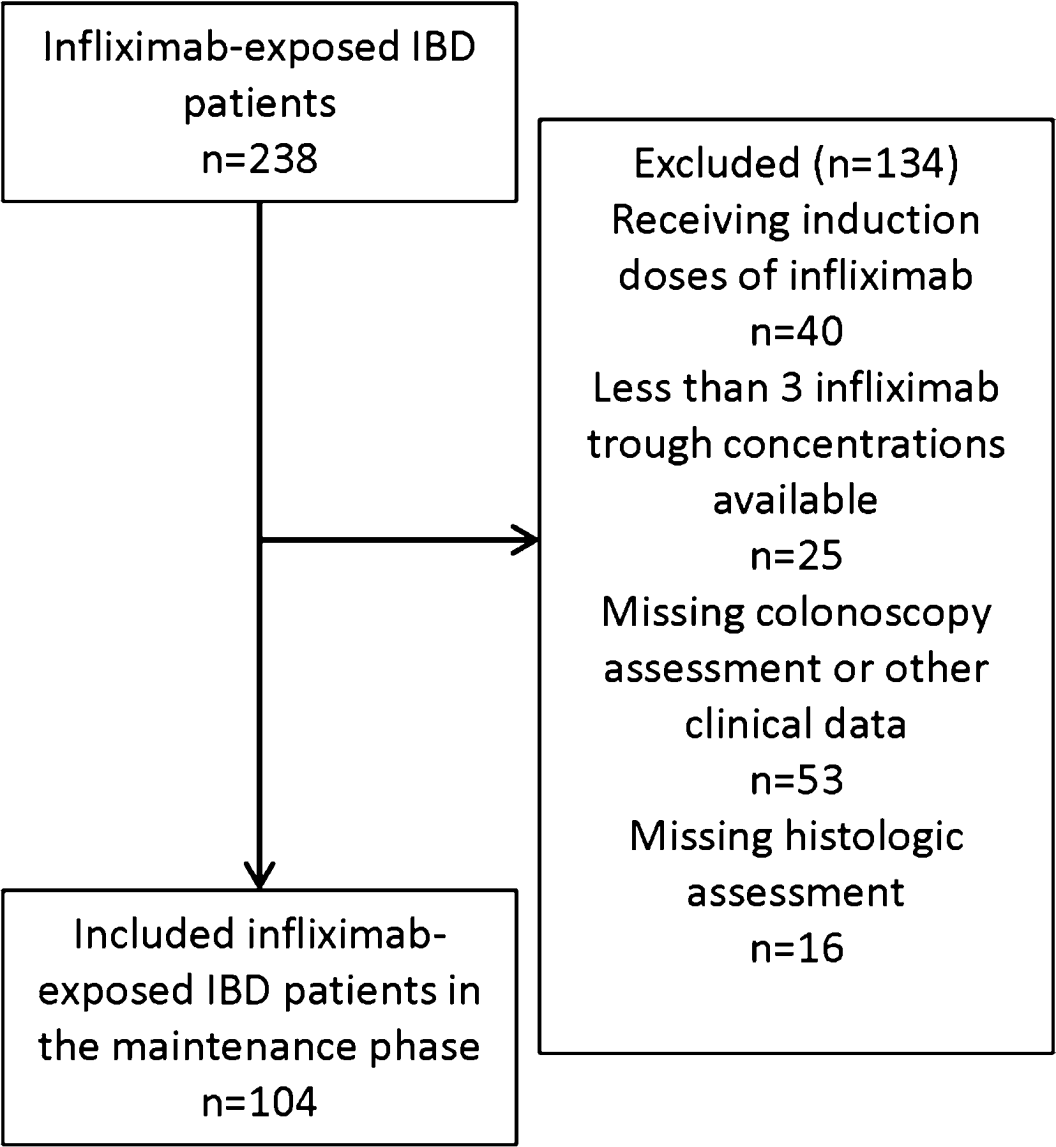
Participant flow through the study. Inflammatory bowel disease (IBD)

A total of 238 patients with IBD were treated with infliximab during the study period. Of those infliximab-exposed patients, 104 were included in this study. The main reason for study exclusion was the absence of one or both colonoscopies or being in the induction phase of infliximab treatment (Fig. 1). Participant characteristics are summarized in Table 1. All participants underwent one colonoscopy during infliximab maintenance phase prior to the period of blood collection. Median time to a follow-up colonoscopy following the period of blood collection was 3 months (IQR 1–7). Median duration of infliximab exposure including exposure pre-enrollment and through the duration of blood collection was 24 months (IQR 12–48). Seventy-eight participants were on high-dose infliximab maintenance therapy at the time of study enrollment. Thirty-seven participants required dose escalation during the course of the blood collection period. All participants provided at least 3 blood samples for infliximab concentration determination. Fifty percent of participants (n = 52) were in histological remission at the start of the observation period while 50% (n = 52) had documented active disease based on histological biopsies. During a median follow-up of 12 months (IQR 6–12), 34.6% (n = 36) participants had sustained histologic remission while 15.4% (n = 16) relapsed, 30.8% (n = 32) had sustained active histologic disease and 19.2% (n = 20) achieved histologic remission. Seventeen participants discontinued infliximab therapy during the period of blood collection due to the loss of treatment response (based on clinical and endoscopic disease activity).

**Fig. 2:**
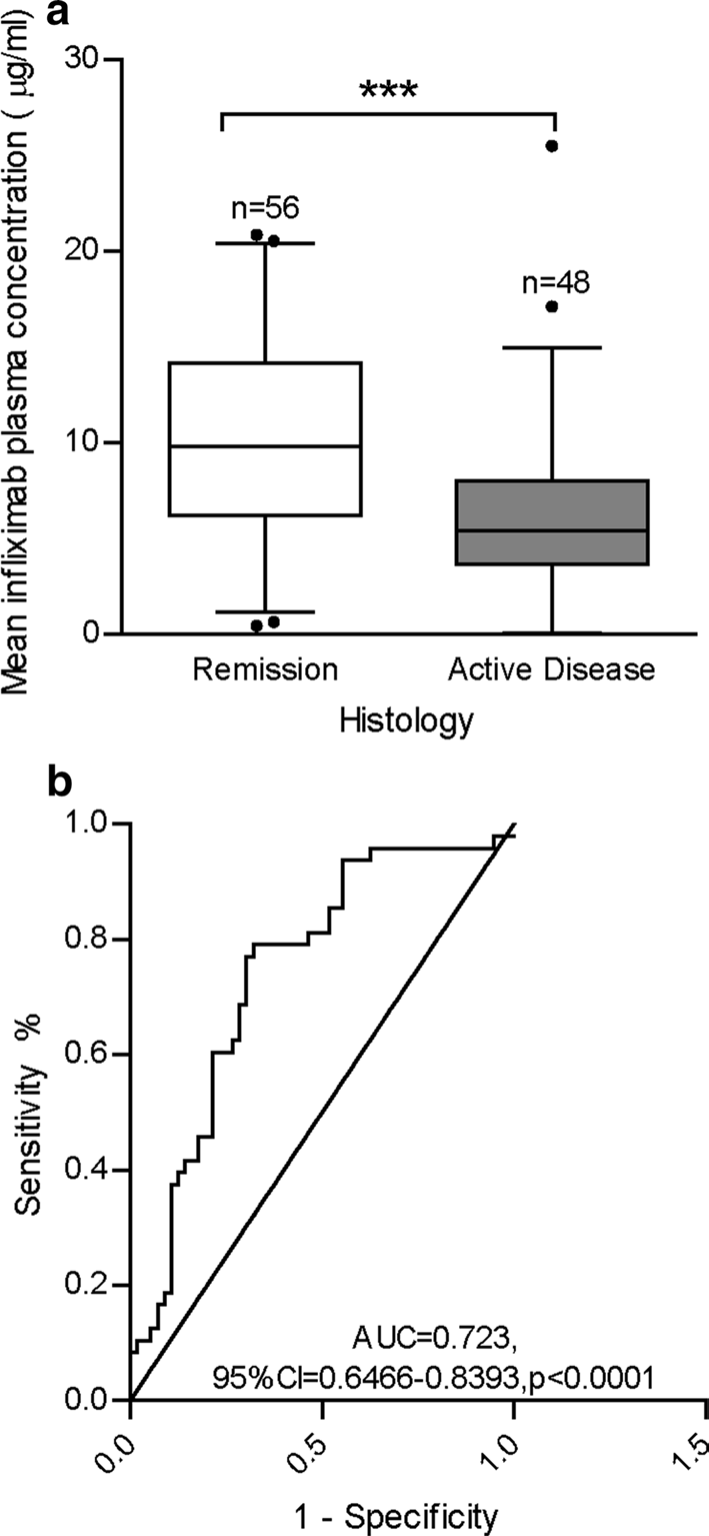
Maintenance-phase mean infliximab trough concentrations in participants with IBD in histologic remission and active histologic disease are represented by box and whisker plot (**a**). Median values (thick horizontal line), 25th and 75th percentile values (box outline), 95% confidence intervals (whiskers); *p < 0.05, **p < 0.01, ***p < 0.001. Receiver operator characteristic analysis for maintenance-phase mean infliximab trough concentrations for participants with and without histologic remission (**b**). Inflammatory bowel disease (IBD); area under the curve (AUC); confidence interval (CI)

Mean infliximab trough concentrations were higher amongst participants who were in histological remission at the end of the study period (10.34 ± 0.69 μg/ml) versus participants who had evidence of active disease (6.23 ± 0.67 μg/ml, p-value < 0.0001) (Fig. 2a). Based on ROC curve analysis (Fig. 2b), a mean infliximab concentration ≥ 8.27 μg/ml (AUROC = 0.723, 95%CI = 0.6466–0.8393, p < 0.0001) was associated with histologic remission (sensitivity = 79.2%, specificity = 67.9%). On multi-variable logistic regression, the odds of achieving histologic remission at the end of the study period was 5 times higher among patients with a mean infliximab trough concentration > 8.27 μg/ml (OR = 5.49, 95% CI = 2.11–14.29, p < 0.0001) (Table 2).

**Table 1:** Participant Demographics

Variables	Total cohort (n = 104)
Age, years (mean, range)	41 (18–74)
Female sex (%)	63 (60.6)
Weight, kg (mean ± std)	78.67 (18.65)
Crohn's disease	68 (65.4)
Ileal	16 (23.5)
Colonic	14 (20.6)
Ileo-colonic	38 (55.9)
Ulcerative colitis	36 (34.6)
Pan-colitis	23 (63.9)
Left-sided colitis	10 (27.8)
Proctitis	3 (8.3)
Median disease duration, years (interquartile range)	6.00 (2.38–11.21)
Smoking history (%)	16 (15.4)
Combination therapy (%)	64 (61.5)
Immunomodulator exposure	
MTX (%)	58 (55.8)
Thiopurine (%)	22 (21.2)
Surgery (%)	29 (27.9)
Anti-infliximab antibodies (%)	9 (8.7)

Kilograms, kg; standard deviation, std; methotrexate, MTX.

**Fig. 3:**
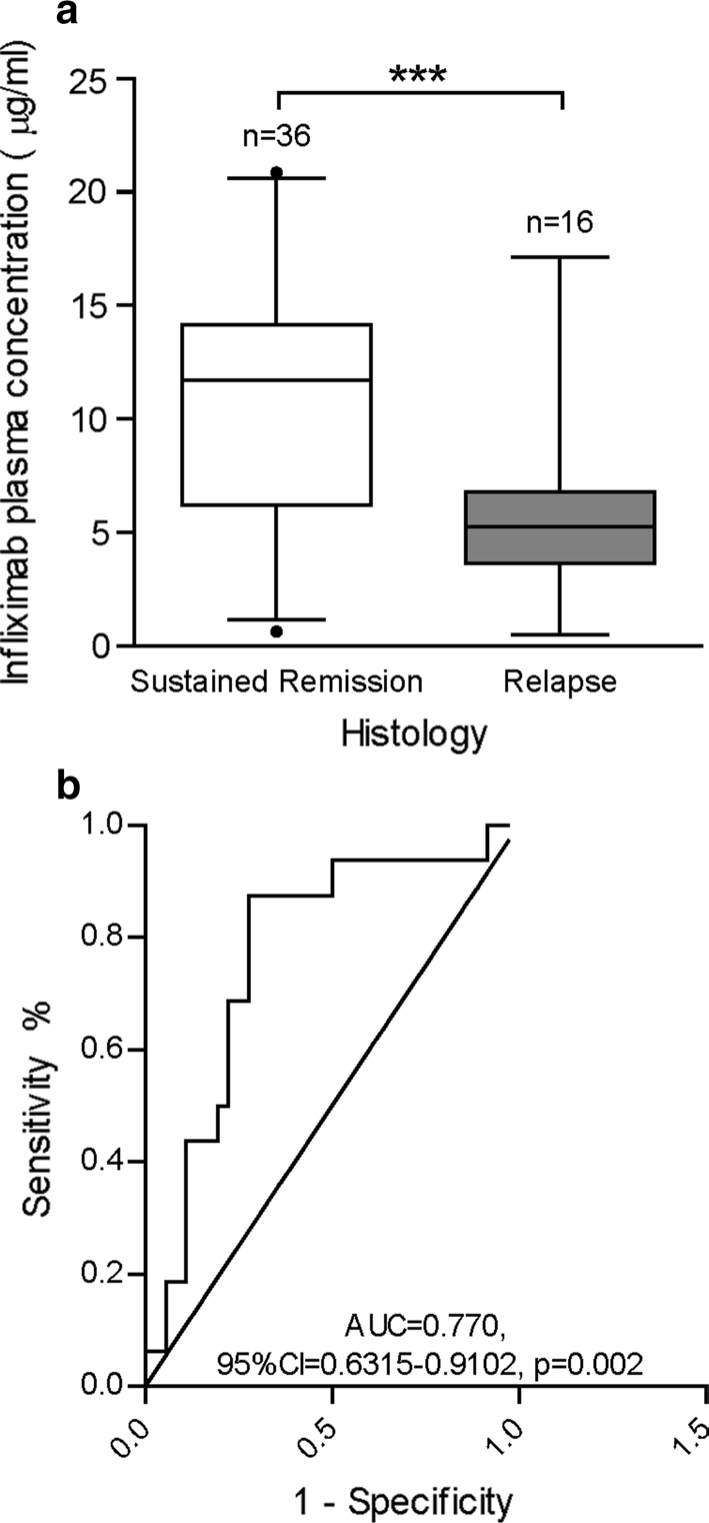
Maintenance-phase mean infliximab trough concentrations in participants with IBD with sustained histologic remission and participants with evidence of histologic relapse are represented by box and whisker plot (**a**). Median values (thick horizontal line), 25th and 75th percentile values (box outline), 95% confidence intervals (whiskers); *p < 0.05, **p < 0.01, ***p < 0.001. Receiver operator characteristic analysis for maintenance-phase mean infliximab trough concentrations for participants with and without sustained histologic remission (**b**). Inflammatory bowel disease (IBD); area under the curve (AUC); confidence interval (CI)

Mean infliximab trough concentrations were higher in participants with sustained histological remission (10.81 ± 5.46 μg/ml) compared to individuals who relapsed while on infliximab therapy (5.68 ± 3.70, p < 0.001) (Fig. 3a). Based on ROC curve analysis (Fig. 3b), a mean infliximab concentration ≥ 8.02 μg/ml (AUC = 0.770, 95%CI = 0.6315–0.9102, p = 0.002) was associated with histologic remission (sensitivity = 87.5%, specificity = 72.2%). On multi-variable logistic regression, the odds of achieving sustained histologic remission was 3 times higher among patients with a mean infliximab trough concentration > 8.27 μg/ml (OR = 3.045, 95% CI = 1.135–8.169, p = 0.027) (Table 3).

**Table 2:** Association between histologic remission and covariates

Covariate	Odds ratio	95% CI	p-value
Age (years)	0.993	0.961–1.027	0.69
Weight (kg)	0.991	0.968–1.015	0.47
Sex	0.814	0.312–2.119	0.69
Diagnosis	1.067	0.382–2.985	0.90
Methotrexate exposure	0.610	0.225–1.656	0.332
Azathioprine exposure	0.861	0.280–2.646	0.793
Presence of anti-infliximab antibodies	0.229	0.033–1.587	0.14
Disease duration (years)	0.955	0.890–1.026	0.18
Concentration > 8.27 μg/ml	5.49	2.11–14.29	< 0.0001

**Table 3:** Association between sustained histologic remission and covariates

Covariate	Odds ratio	95% CI	p-value
Age (years)	0.997	0.963–1.034	0.89
Weight (kg)	0.991	0.966–1.016	0.47
Sex	0.649	0.243–1.735	0.39
Diagnosis (CD or UC)	1.038	0.343–3.143	0.95
Methotrexate co-exposure	0.256	0.086–0.756	0.014
Azathioprine co-exposure	1.050	0.328–3.367	0.93
Presence of anti-infliximab antibodies	0.00	0.000	0.999
Disease duration (years)	0.934	0.854–1.022	0.14
Concentration > 8.27 μg/ml	3.045	1.135–8.169	0.027

Participants who achieved remission during the study period had higher mean infliximab concentrations (9.49 ± 4.60 μg/ml) compared to individuals who relapsed during the study period (5.68 ± 3.70 μg/ml, p < 0.012) and individuals who were secondary non-responders and failed to recapture response throughout the study period (6.51 ± 4.99 μg/ml, p < 0.033). Intra-patient variation in infliximab trough concentrations was significantly higher in participants who relapsed (0.85) or achieved new remission (0.84), while individuals who had a sustained remission had a significantly lower degree of variation in their infliximab trough concentrations over time (0.34, p < 0.0001).

**Fig. 4:**
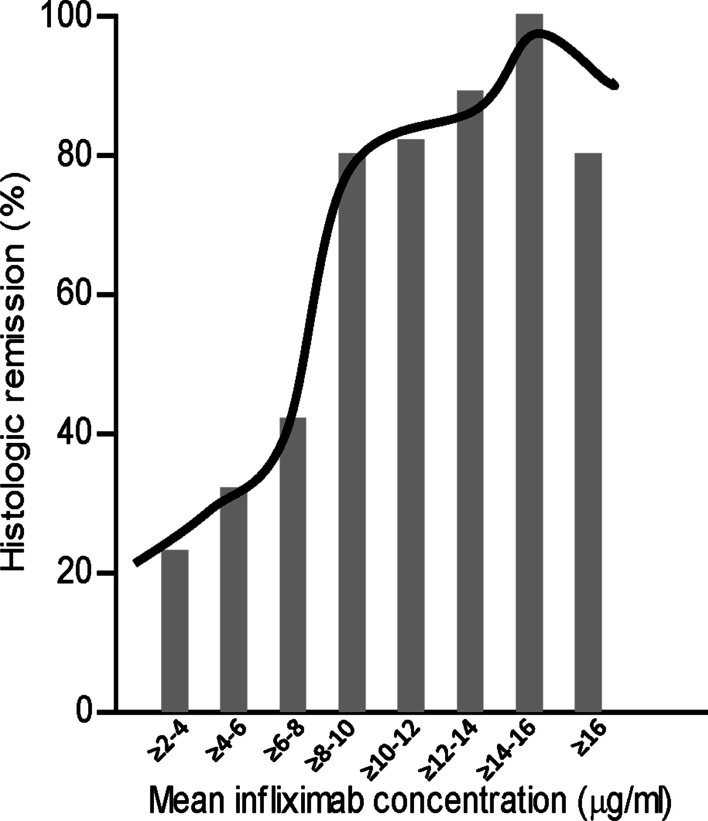
Gain in percent population histologic remission relative to mean infliximab trough concentration

Lastly, an incremental gains analysis was used to assess the incremental increase in histologic remission based on a 2 μg/ml-increase in mean infliximab trough concentration [33]. There was an exponential gain in histologic remission moving from mean infliximab trough concentrations 2 μg/ml (25%) to 8 μg/ml (> 80%) (Fig. 4). No significant gain in histologic remission was seen in this population with mean infliximab trough concentrations > 8 μg/ml.

## Discussion

While a fundamental gap in our knowledge of IBD therapeutics is the inability to predict drug response, we have learned from multiple studies evaluating widely prescribed biologics such as infliximab and adalimumab, that high serum drug concentrations are associated with greater rates of clinical response and remission[29] Targeting adequate anti-TNF blood concentrations, as defined by the AGA, has become part of the routine practice of many gastroenterologists[27] Interestingly, the intended outcomes, ranging from the absence of symptoms to increasingly refined definitions of mucosal healing, remain a topic of discussion. Clinicians are increasingly aware that intestinal healing, specifically histologic remission, is associated with improved long-term outcomes [11, 23, 24] Despite this, there remains a paucity of data on the trough concentrations of infliximab or adalimumab needed to achieve histologic remission.

In this study, we aimed to evaluate the threshold maintenance-phase infliximab concentration associated with histologic remission. We found that a mean infliximab plasma concentration greater than 8 µg/ml in the maintenance phase of treatment was independently associated with histologic remission (8.27 µg/ml) as well as sustained histologic remission over time (8.02 µg/ml). There are several studies evaluating infliximab concentrations and clinical and endoscopic outcomes in IBD[29] Interestingly, there are only two other published studies evaluating infliximab concentration thresholds and histologic remission. In these studies, Papamichael *et.al.* (IBD 2018, APT 2018) demonstrated that a high infliximab concentration in the maintenance phase was associated with histologic remission in CD (> 9.8 µg/ml) and UC (10.5 µg/ml) [9, 34] In both, a point estimate infliximab concentration was assessed within 3 months of endoscopic assessment. Given this absence of longitudinal data, neither study was able to address the question of sustained remission which is of critical importance in chronic illnesses such as these. It is not surprising that higher infliximab trough concentrations are needed to achieve histologic remission. Yarur *et.al.* (2017) showed that significantly higher infliximab trough concentrations, than what are recommended to achieve clinical remission in IBD, are needed to achieve fistula closure in CD [35] This suggests that a higher infliximab exposure is needed to drive change at the level of the microanatomy.

Indeed, to the best of our knowledge, this is the first study to demonstrate an association between infliximab trough concentrations in the maintenance phase and sustained histologic remission over time. There is significant value in knowing the target concentrations associated with maintenance of remission as this outcome is what reduces risk of surgery and other complications related to disease severity.

We additionally performed an incremental gains analysis similar to Ungar *et.al.* (2016) [33] This group was the first to employ this type of analysis in an IBD patient population. They found that infliximab concentrations greater than 8 µg/ml were unlikely to result in further gain in mucosal healing. In our own analysis, we similarly found that the greatest gain in histologic remission was at an infliximab threshold of 8 µg/ml. There was little gain in histologic remission with plasma concentrations greater than 8 µg/ml. The upper threshold infliximab concentration beyond which little gain is seen in patient outcomes is not routinely discussed. As suggested by Ungar *et.al.* (2016), defining the upper limit infliximab concentration has important clinical implications as it could avoid unnecessary dose escalations, in addition to allowing important cost-savings[33]

We also observed that sustained remission is associated with a lack of intra-patient variability in infliximab concentrations over time. This may reflect the true retention of infliximab concentrations within a therapeutic window associated with remission or may be the result of a lack of dose-adjustment by clinicians needing to maximize drug exposure in patients who have a response to treatment.

This study had several limitations. This was an observational design and participants without two colonoscopies were excluded. This may have introduced bias if there were inherent differences between individuals who underwent multiple colonoscopies versus those who did not. Physicians also had the opportunity to adjust dosing of infliximab based on clinical and/or endoscopic outcomes. This approach was not standardized; however, the serial monitoring of infliximab concentrations over time seen herein provides a more accurate representation of the infliximab exposure required for remission than a single blood concentration as is typically reported in most studies of infliximab exposure.

Seventy-five percent of participants entered the study on high dose infliximab and 35% underwent dose escalation during the course of the observation period. This is a higher or proportion of participants receiving high-dose infliximab than has been reported in other populations[36, 37]. This may reflect a more severe IBD patient population or may be a consequence of evaluating participants who are on long-term infliximab and require higher plasma concentrations to achieve histologic remission. Additionally, colonoscopies were performed at different points relative to the collection of blood for infliximab concentration quantification and the median time between colonoscopies was 3 months. This interval between colonoscopies may be too short to assess for full interval changes in day-to-day clinical practice.

Moreover, IBD patients were analyzed in total rather than as UC or CD and no fistulizing patients were included. Differences in disease type or disease phenotypes such as fistulization in CD may require higher infliximab concentrations; however, in studies by Papamichael *et.al.* (2018, 2018), only slightly higher infliximab concentrations were needed in UC versus CD to achieve histologic remission [9, 34] Additionally, the multivariable analysis completed in this study did not identify disease type as a significant modifying factor. Lastly, there is no validated tool for the assessment of histologic remission. Local expertise was used to define histologic remission; however, in future, having a validated tool for histologic activity assessment would make it easier to apply the findings of this study, in addition to others, to the broader IBD population. Additionally and in future, the use of two expert pathologists would serve to further reduce bias. The difficulty in defining histologic remission may be further compounded by the patchy nature of CD, which may have led to missed areas of histologically active disease.

## Conclusion

Ultimately, this study demonstrates that a mean maintenance-phase infliximab concentration greater than 8 μg/ml is associated with histologic remission as well as sustained histologic remission in an IBD cohort. This is higher than what is recommended by current professional guidelines. These data are hypothesis-generating and further study is needed to establish the causality of infliximab concentrations and histologic remission.

## Data Availability

The datasets generated and/or analysed during the current study are not publicly available, but are available from the corresponding author on reasonable request.
